# *Saccharomyces Cerevisiae* Cell Wall Components as Tools for Ochratoxin A Decontamination

**DOI:** 10.3390/toxins7041151

**Published:** 2015-04-02

**Authors:** Małgorzata Piotrowska, Anna Masek

**Affiliations:** 1 Institute of Fermentation Technology and Microbiology, Lodz University of Technology, Wólczańska 171/173, 90-924 Łódź, Poland; 2 Institute of Polymer and Dye Technology, Lodz University of Technology, Stefanowskiego 12/16, 90-924 Łódź, Poland; E-Mail: anna.masek@p.lodz.pl

**Keywords:** ochratoxin A, yeast, adsorption, *Saccharomyces cerevisiae*, glucan, cell wall

## Abstract

The aim of this study was to evaluate the usefulness of *Saccharomyces cerevisiae* cell wall preparations in the adsorption of ochratoxin A (OTA). The study involved the use of a brewer’s yeast cell wall devoid of protein substances, glucans obtained by water and alkaline extraction, a glucan commercially available as a dietary supplement for animals and, additionally, dried brewer’s yeast for comparison. Fourier Transform Infrared (FTIR) analysis of the obtained preparations showed bands characteristic for glucans in the resulting spectra. The yeast cell wall preparation, water-extracted glucan and the commercial glucan bound the highest amount of ochratoxin A, above 55% of the initial concentration, and the alkaline-extracted glucan adsorbed the lowest amount of this toxin. It has been shown that adsorption is most effective at a close-to-neutral pH, while being considerably limited in alkaline conditions.

## 1. Introduction

Ochratoxin A is a nephrotoxic fungal metabolite that contains a chlorinated isocoumarin moiety linked through a carboxyl group to L-phenylalanine via an amide bond. The International Agency for Research on Cancer (IARC) determined it to be a possible human carcinogen (group 2B) [1]. OTA is produced by *Penicillium* species such as *P. verrucosum* and *P. nordicum*, and by *Aspergillus* species such as *A. ochraceus*, *A. melleus*, *A. ostanius* and *A. westerdijkiae*, as well as the *Aspergillus* species of section *Nigri*, e.g., *A. carbonarius*, *A*. *foetidus*, *A*. *lacticoffeatus*, *A*. *niger*, *A*. *sclerotioniger* and *A*. *tubingensis* [2,3,4]. Human exposure to ochratoxin A comes from the consumption of foodstuffs of plant origin (grape juice, wine, coffee, spices, dried fruits, liquorice, chestnuts, cereal-based products, e.g., whole-grain breads), and animal origin, e.g., pork and pig blood-based products [4].

Mycotoxins contamination of food of plant origin and feed can be avoided by taking preventive measures in accordance with the Good Agricultural Practice (GAP) and the Good Manufacturing Practice (GMP), such as the use of appropriate agricultural treatments, e.g., crop rotation, soil cultivation or the use of insecticides. Moreover, post-harvest strategies that should be applied include the improvement of drying and storage conditions by utilizing of chemical, physical or biological methods [5,6]. The approach to prevent the contamination of animal origin food consists of the use of feed of the proper quality and the feed supplementation by adsorbents in order to reduce the absorption of mycotoxins from the gastrointestinal tract and their distribution to blood and target organs [5,7].

The most promising decontamination methods include the use of microorganisms, especially lactic acid bacteria and yeasts, which is the subject of review and original articles [8,9,10,11,12,13,14,15]. The yeasts *Saccharomyces cerevisiae* are widely used in many biotechnological processes in the baking, brewing, winemaking and distilling industries. Due to the frequent mycotoxins contamination of raw materials used in these processes–flour, malt, and grape musts, researchers consider the possibility of conducting fermentation using strains that, in addition to appropriate technological features, have the ability to reduce the content of toxins. It has been shown that certain oenological strains are able to remove ochratoxin A from grape juices and musts [16,17,18,19]. In our previous publications, we have shown that the yeasts, *Saccharomyces cerevisiae*, are characterized by their ability to remove ochratoxin A both from microbiological media and during biotechnological processes such as winemaking [12,13]. In their research Petruzzi and co-workers have demonstrated that the yeast’s adsorption ability in model wine depends on pH, temperature, and ethanol concentration. The best adsorption was observed in 15% of ethanol, pH 3.5, and at a temperature 30 °C [20]. It has been demonstrated that the adsorption of OTA to the surface of both living and dead cells is responsible for the binding process [16]. Heat-inactivated yeast cells of particular strains may be used as adsorbents of mycotoxins in the winemaking process. The using of inactive dry yeasts can improve technological processes, sensory characters and avoid the negative impact of OTA on human health [17]. A cell wall and the charge of the cell wall surface play the main role in the adsorption process [16]. Moreover, the properties of adsorbed toxins namely polarity, solubility, and charge distribution play a significant role in this process [21]. It was shown in the different binding mechanisms, *i.e.*, non-covalent interaction, hydrogen bond, ionic or hydrophobic interaction [22]. The cell wall of the yeasts *Saccharomyces cerevisiae* is composed of polysaccharide fraction (85%–90%) and protein fraction (10%–15%). Mannans and mannoproteins comprise between 30% and 40% of dry weight of yeast, β-1,3-glucan (30%–50%), highly branched β-1,6-glucan (approx. 10%), while chitin content does not exceed 1% [23].

Studies on the adsorption of mycotoxins by yeast cell wall components refer primarily to *Fusarium* toxins, zearalenone and T-2 toxin. Freimund and co-workers [24] showed that crosslinked 1,3-β-d-glucan modified by carboxymethyl ether and hexadecyltrimethylammonium salt demonstrated the highest ability to bind zearalenone (183 mg/g) and T-2 toxin (10 mg/g). Yeasts and their cell wall components are also used as feed additives for animals, and as adsorbents that effectively limit mycotoxicosis in farm animals. The research of Raju and Devegowda [25] suggests that the esterified form of β-d-glucan in a yeast’s cell wall has a protective function in broiler chickens exposed to individual and combined aflatoxin B1, ochratoxin A and T-2 toxin mycotoxicoses. However, research conducted by Baptista and co-workers [26] indicates that manno-oligosaccharides did not suppress damage to animal liver tissue caused by aflatoxins. When administrated intravenously or orally, water-soluble and -insoluble β-glucans stimulate the host immune system, modulate humoral and cellular immunity, and demonstrate anticytotoxic, antimutagenic and antitumoregenic properties. Moreover, they have a beneficial effect on fighting microbial infections [27,28].

The potential application of glucans and other cell wall components as mycotoxin adsorbents from feed depends on the stability of the toxin-cell wall complex under the conditions of the gastrointestinal tract. According to Yiannikouris and co-workers [29] zearalenone adsorption is most effective at acidic and close-to-neutral pH values that prevail in certain regions of the gastrointestinal tract. Petruzzi and co-workers [30] demonstrated that ochratoxin A binding by *Saccharomyces cerevisiae* is reversible and the stability of the OTA-yeast cells complex depends on kind of strains, pH and sugar concentration.

The literature provides some information on the removal of ochratoxin A by dissected yeast cell wall components [22,31]. It has been demonstrated that a mixture of chitin and β-glucan as well as their hydrolysates have the ability to remove from 64% to 74% of ochratoxin A from wine contaminated by OTA (5 μg/L) [32].

The aim of this study was to evaluate the ability of preparations obtained from dried brewer’s yeast, such as the cell wall, glucans extracted by two methods and a commercially available glucan to adsorb ochratoxin A. The effect of pH on the effectiveness of this process was also investigated.

## 2. Results and Discussion

**Figure 1:**
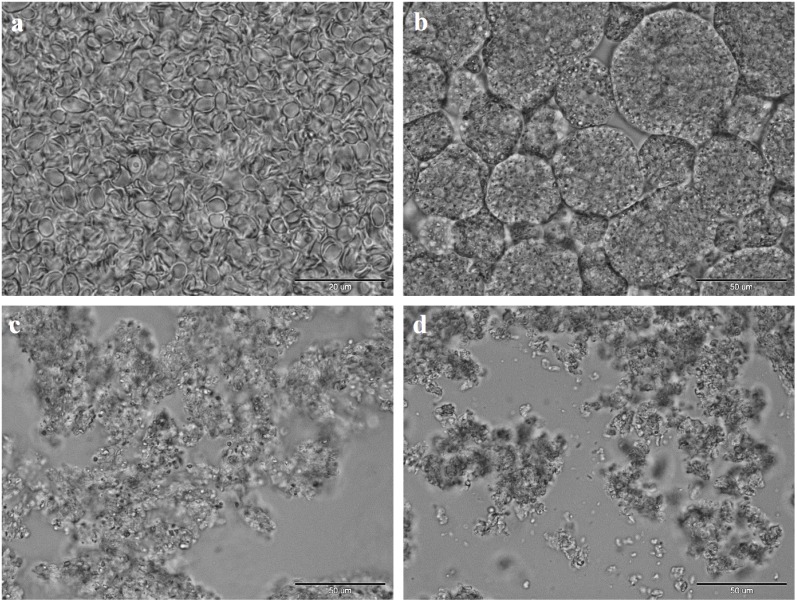
Optical micrographs of cell wall and glucan samples (**a**) yeast cell walls CW; (**b**) commercial glucan CG; (**c**) alkaline extracted glucan AG; (**d**) water extracted glucan WG; bar 20 µm (**a**) and 50 µm (**b**–**d**).

During the study, three dried brewer’s yeast preparations were obtained: a cell wall (CW) and glucans extracted from cell walls by the action of alkali (AG) and water (WG). A commercially available glucan (CG), which according to the manufacturer’s declaration has more than 70% of (1,3)-(1,6)-β-d-glucan, was also tested. Figure 1 shows microscopic images of the investigated preparations. Figure 1a shows the yeast cell wall devoid of protein substances; the cell shape is retained but it is devoid of the internal organelles. The commercial glucan forms regular conglomerates with a diameter of 10 to 70 µm. The studies of commercial available products show that they have mainly globular structures of 5 to 100 µm. However, it is desirable that such preparations have a form of smaller particles that have a higher immunological activity especially in the activation of macrophages and are more useful as additives to pharmaceutical or cosmetic preparations [33]. There are no visible morphological differences between the alkaline- and water-extracted glucans in the photomicrograph. The visible conglomerates are not as regular as the CG (Figure 1c,d).

**Figure 2:**
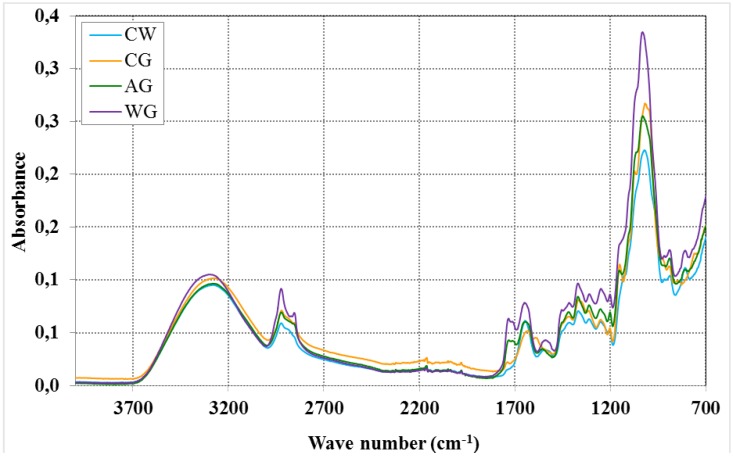
FTIR spectra of yeast-derived products CW–cell walls; CG–commercial glucan; AG–alkaline extracted glucan; WG–water extracted glucan.

There were no significant differences between the samples in the FTIR spectra (Figure 2). Two spectrum regions are characteristic of polysaccharides: “the sugar region” (950–1200 cm^−1^) and “the anomeric region” (750–950 cm^−1^) [34,35,36]. The FTIR spectra showed a similar spectral pattern for all samples, typical of a β-1,3-glucan with carbohydrate adsorbing regions. There were only differences in the intensity of absorbance: the cell walls (CW) showed the lowest intensity. A band at 1420 cm^−1^ was reported in each sample of the glucans (AG, CG, WG), indicating the presence of β-glucan. The absence of a band at 910 cm^−1^ indicates the lack of α-glucan configuration. The band around 3300 cm^−1^ indicates OH stretching vibration of hydroxyls [36].

Protein residues were recorded in the samples, as evidenced by the bands at 1400–1700 cm^−1^, and they were of greater intensity in the alkaline- and water-extracted glucans. These peaks indicate the presence of chitin, a minor component of the yeast cell wall, and probably some products of protein degradation [36].

**Table 1:** The ochratoxin A (OTA) adsorption ability of the yeast-derived products.

Adsorbent	Ochratoxin A Adsorption (%)
Dried brewery yeast (DY)	41.63 ± 0.64^a^
Cell wall (CW)	57.83 ± 1.03^b^
Glucan–alkaline extraction (AG)	25.53 ± 1.92^c^
Glucan–water extraction (WG)	55.22 ± 1.06^d^
Commercial glucan (CG)	56.37 ± 0.67^db^

Note: Values in the table represent means from three samples ±SD; different letters in columns designate statistically significant differences (one-way ANOVA, *p* < 0.05).

Table 1 shows the results of ochratoxin A adsorption by a variety of cell wall preparations—the derivatives and the dried yeast for comparison. The yeast cell wall preparation, water-extracted glucan and the commercial glucan bound the highest amount of ochratoxin A, above 55% of the initial concentration (*p* < 0.05). The alkaline-extracted glucan bound the lowest amount not exceeding 30%. There were no differences in adsorption properties between the water-extracted glucan and the commercial glucan (*p* > 0.05). Our previous studies have shown that in a suspension of the same density, living cells of the bakers’ yeasts, *Saccharomyces cerevisiae*, adsorbed OTA to an extent not exceeding 30%, *i.e.*, 59.4 ng/mg of the adsorbent, with the same starting content [12]. Yiannikouris and co-workers [37] have shown that β-(1,3 and 1,6)-d-glucans and related alkaline-extracted fractions isolated from the cell wall of *Saccharomyces cerevisiae* are able to adsorb zearalenone *in vitro* with an affinity of up to 50%. Other *in vitro* studies have demonstrated that the yeast cell wall is capable of binding zearalenone (66.7%), fumonisin (67.0%), DON (12.6%), citrinin (18.4%), T-2 toxin (33.4%) and DAS (12.7%) [38]. According to Joannis-Cassan and co-workers [39] the cell wall from baker’s yeast can adsorb up to 62% of OTA depending on the mycotoxins concentration and yeast composition.

The preparations were devoid of protein substances, while the remaining traces were detected by FTIR. It can therefore be concluded that the polysaccharide fraction of the yeast cell wall, namely β-glucans, is responsible for the adsorption of ochratoxin A. This is confirmed by the studies of Caridi and co-workers [8] and Ringot and co-workers [31].

Very low OTA adsorption by glucans obtained by KOH extraction, which contain alkali-insoluble glucans, can result from changes in the conformation of the glucans during extraction and different composition of sugars compared to the other preparations. The alkali-insoluble glucans isolated from *Saccharomyces cerevisiae* consisted mostly of glucose (89%) and small amounts of mannose (2%) and *N*-acetylglucosamine (9%) [23].

**Figure 3:**
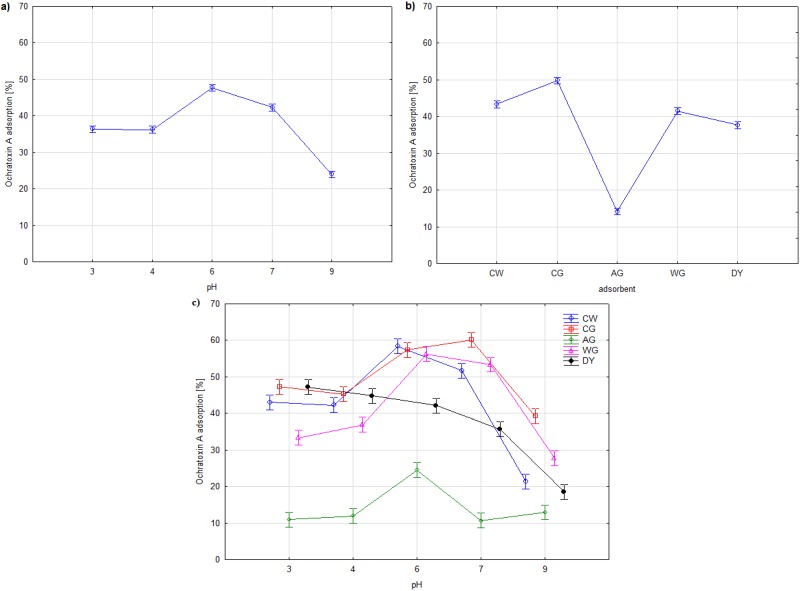
Two-way ANOVA for the effects of pH (**a**); kind of adsorbent (**b**); pH *vs.* kind of adsorbent (**c**) on ochratoxin A adsorption. Vertical bars denote 95% confidence.

In Figure 3 the result of two-way ANOVA analysis is presented. The analysis of variance shows the significant influence of the pH, the kind of adsorbent and their interactive effect on ochratoxin A adsorption (Table 2).

**Table 2:** Effects of the pH and kind of adsorbent on the ochratoxin A adsorption.

Factor	Sum of Squares SS	Degrees of Freedom df	Mean Square MS	*F*	*p*
Adsorbent	11,184.017	4	2796.004	913.499	<0.001
pH value	4688.358	4	1172.090	382.940	<0.001
Adsorbent × pH value	2443.524	16	152.720	49.896	<0.001
Error	153.038	50	3.061		

Note: Two-way ANOVA, (*p* < 0.05).

It was shown that the adsorption of ochratoxin A was the highest in the close-to-neutral pH range of 5.5 to 7 (Figure 3). There were no significant differences in the amount of adsorbed toxin by the isolated cell wall (CW), commercial (CG) and water extracted glucans (WG). The amount of ochratoxin A adsorbed in this pH range exceeded 55% of its initial content. Lower adsorption was observed in the case of the dried yeast and the KOH-extracted glucan. Faucet-Marquis and co-workers [40] demonstrated the best adsorption of OTA by a yeast cell wall in pH 3, up to 60% of initial concentration in the range of 0.5–10 µg/mL.

An increase in pH above 8 significantly reduced the adsorption properties, while the lowest reduction was observed in the case of the commercial glucan. The amount of adsorbed toxin ranged from 13% to 28%.

A similar trend was observed by Yiannikouris and co-workers [29] in the study of zearalenone adsorption. They found that acidic and neutral conditions gave the highest affinity rates (64% to 77%) by β-(1,3)-d-glucans. It was also noted in [39] in relation to zearalenone, aflatoxin B1 and ochratoxin A. As in our study, alkaline conditions decreased adsorption. This may be due to changes in the conformation of glucans in alkaline conditions. The adsorption process is dependent on the three-dimensional organization of β-glucans. Alkaline conditions destabilize their spatial organization and favour single helix and/or random coil structures [29,41]. Moreover, in the high pH, above 8 modification of the ochratoxin A molecule can occur. The open of the lactone cycle of OTA was observed [42,43].

## 3. Experimental Section

### 3.1. Biological Material

The experiments involved the use of dried brewer’s yeasts *Saccharomyces cerevisiae* (DY) obtained from InterYeast–d (InterYeast, Krośniewice, Poland).

### 3.2. Commercial Glucan

A glucan preparation designed as a feed additive, Leiber Biolex^®^-Beta S (InterYeast, Krośniewice, Poland), was also used in the study.

### 3.3. Chemicals

All chemicals and solvents used were purchased from SIGMA-Aldrich, (St. Louis, MO, USA) and were of analytical grade. Water from a Milli-Q system (Millipore, Billerica, MA, USA) was used for all solutions, dilution and the mobile phase for HPLC. Ochratoxin A was stored as a stock solution in absolute ethanol (HPLC grade) at −20 °C. The concentration of OTA in stock solution was 200,000 ng/mL.

### 3.4. Isolation of the Yeast Cell Wall

The cell wall was isolated based on methods proposed by Nguyen and co-workers [22] and Liu and co-workers [44] with some original modifications. Ten grams of glass beads (diameter of 0.75–1.0 mm) were added to the same amount of dried yeast, and this was filled up with 10 mM phosphate buffer (pH 8.0) to a volume of 50 mL. The mixture was shaken at 30 °C for four hours. The degree of cell disruption was monitored in a microscope. Glass beads were then separated by decantation, and the cell suspension was centrifuged (5000× *g*, 15 min) and washed twice with a phosphate buffer. In order to separate protein substances, 1% SDS (sodium dodecyl sulphate) was added to the remainder and shaken for three hours at 30 °C. The washing out of the protein substances was monitored by measuring the absorbance of the supernatant at 260 nm (DU 640 spectrophotometer, Beckman Coulter, Inc., Brea, CA, USA). The resulting cell walls preparation was dried in absolute ethanol.

### 3.5. Glucan Fraction Preparation from the Yeast Cell Wall

Two methods were used: alkaline extraction and water extraction. The obtained cell wall preparation was subjected to extraction with 1 M KOH at 4 °C for 20 h with gentle agitation according to Nguen and co-workers [23]. The mixture was then centrifuged and the pellet was washed several times with water until a neutral pH was reached. An alkali-insoluble glucan fraction was found in the precipitate, which after drying in the air was used in further studies.

In the second method, the cell wall preparation was treated with water at 121 °C for four hours (in an autoclave) according to the method proposed by Liu and co-workers [44] and Freimund and co-workers [45]. The water-insoluble fraction was then centrifuged, air-dried and used in further studies.

### 3.6. Ochratoxin A Adsorption by the Yeast Cell Wall Preparation

The experiment involved the use of a glucan obtained by water (WG) and alkaline (AG) extraction, commercial glucan (CG), and cell wall preparations (CW). Dried brewer’s yeast (BY) was also used for comparison.

Ochratoxin A removal by the adsorbents was studied in 0.1 M PBS (phosphate-buffered saline) pH 6.2. The influence of the pH value on OTA adsorption was examined in 0.1 M phosphate buffer adjusted to pH 3, 4, 6, 7 and 9. Both media were contaminated with OTA, the initial OTA concentration 1000 ng/mL. The buffer was inoculated with glucans and the initial concentration was 5 mg of dry weight (dw)/mL. The suspension density was standardized with the turbidimetric method by using T60 UV-Visible Spectrofotometer (PG Instruments, Lutterworth, UK) at 540 nm. The standard curve of absorbance as a function of dry biomass amount was used in the calculations of density of yeasts biomass [46]. Incubation was conducted with agitation (200 rpm) at 30 °C for 24 h. After 24 h, the samples were centrifuged at 7000× *g* for 10 min and the amount of residual OTA in the supernatant was determined. The control sample consisted of the same mixtures, but without adsorbents. In the control sample the OTA concentration at the beginning and the end of experiments was determined too. The reduction of OTA content was calculated relative to the value in control samples and expressed in a percentage.

### 3.7. Ochratoxin A Determination

The samples were extracted and cleaned up using an OchraStar^®^ immunoaffinity column (Romer Labs^®^ Diagnostic GmbH, Tulln, Austria) according to producers’ instruction. Ochratoxin A was determined by HPLC methods using a Finnigan™ Surveyor Plus™ chromatograph (Thermo Separation Products, Riviera Beach, FL, USA) with an Ace 5 μm C18 column (250 mm × 4.6 mm), an Ace 5 C18 guard column (Advanced Chromatography Technologies, Aberdeen, UK), a loop 50 μL, at a flow rate of 1 mL/min, at an ambient temperature, with water: acetonitrile: glacial acetic acid (99:99:2, *v*/*v*/*v*) as the mobile phase, fluorescence detection (λ_excitation_ = 330 nm, λ_emission_ = 460 nm).

### 3.8. Optical Microscopy

In order to observe the microstructure of obtained preparations (the presence of conglomerates and size of particles) an Olympus CX41 optical microscope (Olympus Life Science Europa GmbH, Hamburg, Germany) and Cell B software (Olympus Digital Imaging Solutions) were used.

### 3.9. Infrared Spectroscopy

In order to assess the structure of examined preparations the FTIR method was used. Infrared spectra were measured within the wavelength range of 3000–700 cm^−1^ using a FTIR Nicolet 6700 spectrophotometer and OMNIC 3.2. software (Thermo ScientificProducts, Riviera Beach, FL, USA). The samples were pressed into KBr pellets with glucan/KBr ratio of 2/200 mg. Fourier transform infrared spectroscopy was performed with a DGTS/KBr detector, with the following measurement parameters: 128 scans, resolution 8 cm^−1^, and the speed of scanning equal to 0.6329 cm/s.

### 3.10. Statistical Analysis

The presented results are mean values from three independent experiments. A statistical analysis (means, standard deviation) and the analysis of variance (one-way and two-way ANOVA). When a statistical difference was detected (*p* < 0.05), means were compared by the *post hoc* Tukey’s test at significance level 0.05. Tests were conducted using ORIGIN, v.6.1 (Microcal, Northampton, MA, USA), 2000 and *Statistica*, v.10.0 (StatSoft, Inc., Tulsa, OK, USA; 2011 software).

## 4. Conclusions

The yeast (*Saccharomyces cerevisiae*) preparations: the cell walls, the glucans obtained by alkaline and water extraction as well as the commercially available glucan preparation have the ability to adsorb OTA. Alkali-insoluble glucans show the lowest ability in this respect. Assessing the effect of pH on the effectiveness of adsorption, it has been found that this process occurs in the highest degree at a close-to-neutral pH, while alkaline conditions greatly reduce the ability of the glucan preparations to adsorb OTA. The results show that the polysaccharide components of the yeast cell wall are responsible for the adsorption of ochratoxin A. The phenomenon of ochratoxin A adsorption by cell wall preparations may be used in practice, e.g., in oenology or as a dietary supplements for human or animals.
